# Supplementary material for: Cost Function Analysis Applied to Different Kinetic Release Models of Arrabidaea chica Verlot Extract from Chitosan/Alginate Membranes
Source: Polymers (Basel). 2022 Mar 10;14(6):1109. doi: 10.3390/polym14061109 (PMC8956060; doi:10.3390/polym14061109)
Supplement: Supplementary file 1 [file polymers-14-01109-s001.zip › SupMat.pdf]

polymers

Article

Cost Function Analysis Applied to Different Kinetic Release Models of *Arrabidaea chica* Verlot Extract from Chitosan/Alginate Membranes

Luis Concha [1], Ana Luiza Resende Pires [2], Angela Maria Moraes [2], Elizabeth Mas-Hernández [3,4], Stefan Berres [5,*] and Jacobo Hernandez-Montelongo [1,4,*]

1 Department of Physical and Mathematical Sciences, Catholic University of Temuco, 4813302 Temuco, Chile
2 School of Chemical Engineering, University of Campinas, 13083-852 Campinas, Sao Paulo, Brazil
3 Department of Mathematical Engineering, University of La Frontera, 4811230 Temuco, Chile
4 Bioproducts and Advanced Materials Research Nucleus (BioMA), Catholic University of Temuco, 4813302 Temuco, Chile
5 Department of Information System, University of Bio-Bio, 4051381 Concepcion, Chile
* Correspondence: jacobo.hernandez@uct.cl (J.H-M.), stefan.berres@gmail.com (S.B.)

Citation: Concha, L.; Resende Pires, A.L.; Moraes, A.M.; Mas-Hernández, E.; Berres, S.; Hernandez-Montelongo, J. Cost Function Analysis Applied to Different Kinetic Release Models of *Arrabidaea chica* Verlot Extract from Chitosan/Alginate Membranes. *Polymers* **2022**, *12*, 1109. https://doi.org/10.3390/polym14061109

Academic Editors: Yadir Torres Hernández, Ana María Beltrán Custodio and Manuel Félix Ángel

Received: 8 December 2021
Accepted: 17 January 2022
Published: 10 March 2022

Publisher's Note: MDPI stays neutral with regard to jurisdictional claims in published maps and institutional affiliations.

Copyright: © 2022 by the authors. Licensee MDPI, Basel, Switzerland. This article is an open access article distributed under the terms and conditions of the Creative Commons Attribution (CC BY) license (https://creativecommons.org/licenses/by/4.0/).

1. Supplementary Materials

The pseudo-code of the calculation of the cost function is described in Algorithm S1. The global computational variables $\hat{\mathbf{t}}$, $\hat{\mathbf{u}}$ contain the measurement data. The computational variable $\mathbf{p}$ contains the model parameters.

Algorithm S1 Cost function

function c = costfun($\mathbf{p}$)
global $\hat{\mathbf{t}}$, $\hat{\mathbf{u}}$
$\mathbf{u}$ = uModel($\hat{\mathbf{t}}$);
c = $\sum_i (u_i - \hat{u}_i)^2$

The subroutine $\mathbf{u}$ = uModel($\hat{\mathbf{t}}$), is evaluated for given times that agree with the measurement times, and is specified in Algorithm S2.

Algorithm S2 Pseudo-code for the implementation of the model function

function $\mathbf{u}$ = uModel($\mathbf{t}$)
global $\mathbf{p}$
$\mathbf{u} = p(1) * \mathbf{t}.\hat{}\,(p(2))$

The computational parameter $\mathbf{t}$ might be a vector, such that the corresponding model values might be calculated simultaneously with results stored in $\mathbf{u}$. By convention, the MATLAB symbol .^denotes a component-wise operation of calculating an exponent. The model parameters $\mathbf{p}$ are maintained as global variables. In this implementation example, the Korsmeyer-Peppas model is taken as reference model function.

In Algorithm S3 the residual is defined as a difference of the model and the data; its input variables are the same as for the cost function, namely the model parameters $\mathbf{p}$ and the data. Reversely, the cost function can be calculated as the 2-norm of the residual.

Algorithm S3 Residual function

function $\mathbf{r}$ = residual($\mathbf{p}$)
global $\hat{\mathbf{t}}$, $\hat{\mathbf{u}}$
$\mathbf{u}$ = uModel($\hat{\mathbf{t}}$);
$\mathbf{r} = \mathbf{u} - \hat{\mathbf{u}}$

In Algorithm S4 the steps of the overall procedure are indicated.

Algorithm S4 Central routine that calls the optimization algorithms

✓ Clear registers, close the windows
✓ Load the observation data
✓ Choose initial optimization parameters
✓ Run the optimization algorithm
✓ Visualize the performance of the optimization algorithms

For didactic reasons, we remind the method of Gauss-Newton, see Algorithm S5; this method is used for referential comparisons to other methods; more sophisticated methods starting with the Levenberg-Marquardt method can deal better with more ill-posed situations.

Algorithm S5 Gauss-Newton method

```
p* ← Gauss-Newton(p0)
global XX FF
XX=[X];
FF=[norm(X)];
for i = 1; i ≤ 10 do
   F ← residual(X);
   J ← jacobian(X);
   DX = -J \ F;
   X=X+DX;
   FF=[FF norm(F)];
   XX=[XX X];
   if norm(F) < TOL then
      break
   end if
end for
```

Global optimization methods or machine learning algorithms might be alternatives to experiment with in order to generate more robust and diverse parameter fitting framework.
