# Supplementary figures and images for: Cost Function Analysis Applied to Different Kinetic Release Models of Arrabidaea chica Verlot Extract from Chitosan/Alginate Membranes
Source: Polymers (Basel). 2022 Mar 10;14(6):1109. doi: 10.3390/polym14061109 (PMC8956060; doi:10.3390/polym14061109)

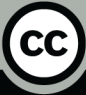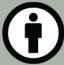

BY

Supplement: Supplementary file 1 [file polymers-14-01109-s001.zip › Definitions/logo-ccby-eps-converted-to.pdf]

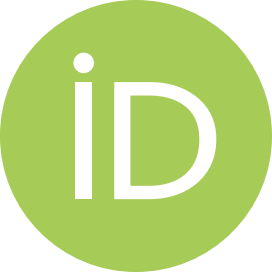

Supplement: Supplementary file 1 [file polymers-14-01109-s001.zip › Definitions/logo-orcid-eps-converted-to.pdf]

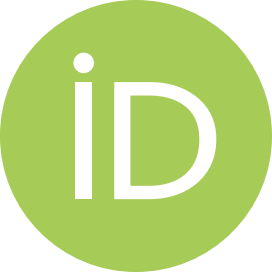

Supplement: Supplementary file 1 [file polymers-14-01109-s001.zip › Definitions/logo-orcid.pdf]

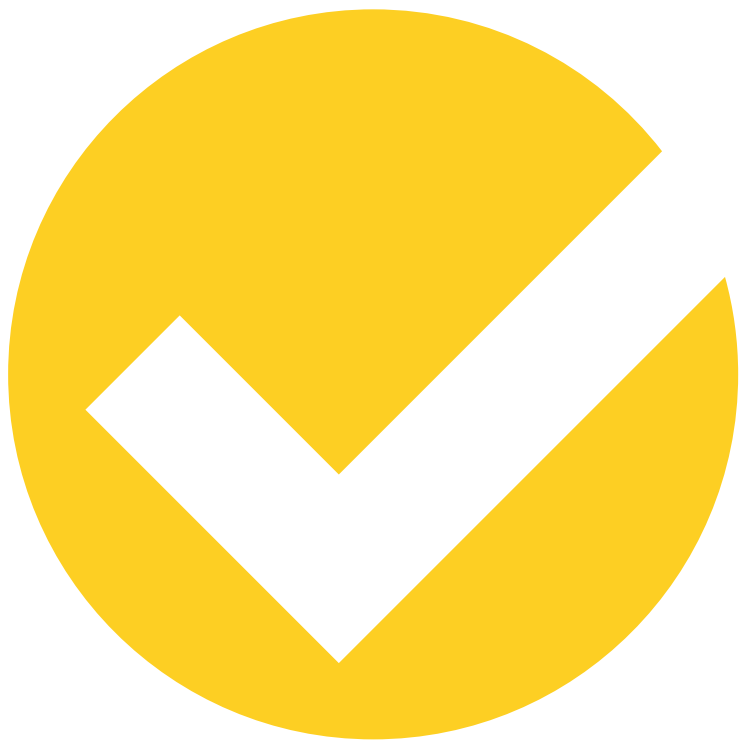

check for  
updates

Supplement: Supplementary file 1 [file polymers-14-01109-s001.zip › Definitions/logo-updates.pdf]

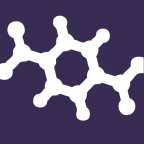

*polymers*

Supplement: Supplementary file 1 [file polymers-14-01109-s001.zip › Definitions/polymers-logo-eps-converted-to.pdf]

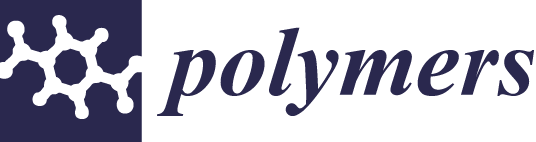

Supplement: Supplementary file 1 [file polymers-14-01109-s001.zip › Definitions/polymers-logo.png]
